# Retraction of “*Toxicity of Nanoscaled Zero-Valent Iron Particles on Tilapia, Oreochromis mossambicus*”

**DOI:** 10.1021/acsomega.6c10641

**Published:** 2026-10-16

**Authors:** Arivarasan Vishnu Kirthi, Gaurav Kumar, Gaurav Pant, Manu Pant, Kaizar Hossain, Akil Ahmad, Mohammed B. Alshammari

The authors retract “Toxicity of Nanoscaled Zero-Valent Iron Particles on Tilapia, *Oreochromis mossambicus*” (DOI: 10.1021/acsomega.2c05696) because there is overlap between panels A, B and C in Figure 4, indicating that the images were taken from the same sample. The authors provided image files during the post-publication review process; however, sufficient confidence in the relationship of the data to the results reported in the article was not assured. This undermines the reliability of the results presented. As such, the article is being retracted.

The original article was published on December 12, 2022, and retracted on October 16, 2026.

